# Helping the medicine go down: the role of the healthcare professional in a young person’s experience of achalasia, a rare oesophageal motility disorder

**DOI:** 10.1186/s13023-025-03571-0

**Published:** 2025-02-20

**Authors:** Geena Capps

**Affiliations:** Medical Sciences Division, University of Oxford, Oxford, UK

**Keywords:** Rare disease, Patient perspective, Mental health, Achalasia, Student

## Abstract

Young patients can be uniquely vulnerable to the impacts of a rare disease, diagnosed in their critical years of identity formation, social development, and planning for the future. Drawing from my journey as both a rare disease patient and a medical student, this essay explores how the rare disease achalasia has shaped my life, alongside the experiences of another young patient, Isobel. Most importantly, this essay highlights the critical role that individual healthcare professionals play in shaping young patients’ experiences of their condition. Although diagnosing and managing rare diseases can be challenging due to limited research and awareness, my own experiences demonstrate that individual, intentional changes can have profound impacts. By engaging with and believing young patients, individual healthcare providers can reduce misdiagnoses, alleviate isolation and uncertainty, and ultimately, improve healthcare outcomes for young people with rare diseases.

## Introduction

“I promise I’m not trying to throw up, Mum,” I pleaded, choking back tears as we left yet another doctor’s appointment. I was 13 and had visited the doctor because I physically could not swallow food, throwing up every time I ate. I had just been misdiagnosed with an eating disorder. Whilst this was not my first, or last, misdiagnosis, this moment marked the point in the journey toward my rare disease’s diagnosis that I had finally given up.

It took 17 months for me to be diagnosed with achalasia, a rare oesophageal motility disorder that occurs due to failure of lower oesophageal sphincter relaxation and affects 1 in 100,000 people annually [1, 2]. Achalasia has had and continues to have significant impacts on my life, physically, socially, and emotionally. In this essay, I explore these impacts, drawing comparisons with another achalasia patient, Isobel, who was similarly diagnosed during her teenage years. Through our experiences, I underscore the power held by healthcare professionals to transform the experience of rare disease (RD) patients through simple, transformative changes: validating our experiences, supporting us holistically, and guiding us into uncertain futures.

## The diagnostic odyssey: facing doubt, losing hope

At 13, I began to notice food feeling “stuck” after eating, writhing to help it ‘go down’. Soon, I was regurgitating food and liquids, and waking up to vomit on my pillow, having regurgitated food even at night. Despite trying an exclusion, then a liquid diet, my symptoms did not resolve. I was told it was stress, and then, an eating disorder. Only after two endoscopies and a barium swallow, was the word “achalasia” mentioned.

Isobel first visited the doctors to complain of pain while eating. She was brushed off as ‘stressed’ and sent home to rest. After weeks of escalating symptoms, unable to swallow anything and losing almost three stone, she visited the emergency department. Here, she was further dismissed: one doctor laughed, asking “what do you expect me to do in A&E?”. Thanks to her persistence, Isobel was eventually admitted to hospital, but even then, the doctors believed her symptoms were psychological. In a tiny room, designated for children with eating disorders, she turned to her father, exhausted, and said, “Dad, they don’t believe me”.

Our shared experience mirrors that of many other RD patients, who endure this prolonged ‘diagnostic odyssey’, lasting, on average, 4.7 years, with women and younger patients facing prolonged diagnosis times [3]. While delayed diagnoses have significant physical impacts, their psychological tolls are also devastating. Notably, individuals presenting with medically unexplained symptoms often face doubts regarding their legitimacy [4]. Physicians may misattribute symptoms to psychological causes, such as stress or, in our case, eating disorders: in a recent survey, 60% of RD patients reported receiving a psychological misdiagnosis [3]. This has substantial repercussions: psychological diagnoses can prolong the time taken to reach a correct diagnosis by 2.5–14 times, depending on the disease [5]. This stigmatizing dismissal has serious implications: afraid to burden my friends and family by my possibly self-inflicted condition, I withdrew from social situations, isolating myself. Had it not been for the incredible support structure around me, I may have given up fighting for a diagnosis, believing that I was at fault for my symptoms.

Difficulties in diagnosis are understandable: there are more than 7000 rare diseases, with diverse presentations often appearing similar to more common conditions, such as achalasia sharing symptoms with eating disorders [6]. Despite this, primary care physicians can transform the diagnostic odyssey, even if they cannot end it, by believing patients, validating their experiences, and commissioning further investigations. The turning point in my diagnosis was meeting a doctor who despite initially suggesting I had an eating disorder still referred me for an endoscopy. Isobel’s path was more tortuous: she begged her nurses to watch her eat, and upon seeing her regurgitate food without any assistance, they referred her onwards for the barium swallow that led to her diagnosis. Without this relentless self-advocacy, her diagnosis might have been further delayed, with devastating physical consequences.

## Receiving a diagnosis: conflicting experiences

I received my diagnosis at school, discovering via email that I had achalasia, a disease I had never heard of. I was told that there was no cure, only management, and that my consultant was “confident there was a 90% chance in relieving my symptoms”. Similarly, Isobel received her diagnosis alone in a hospital room, without her parents, an audience of students observing as her doctor casually “threw out” her diagnosis. These experiences are not isolated incidents: many RD patients find the process of receiving a diagnosis stressful, feeling uninformed and unsupported psychosocially after the diagnosis [7].

However, at another hospital, Isobel had a much more positive experience: despite visiting hours being over, her parents were allowed to visit. Her consultant was compassionate, carefully outlining her next treatment steps and encouraging her to ask questions, although acknowledging that he may not have all the answers. Hence, diagnosis delivery is a critical opportunity for a positive healthcare experience: a good diagnosis is clearly communicated, delivered sensitively, and in the correct environment.

Diagnosis delivery is a starting point, not an end-point: as with many other rare diseases, achalasia cannot be cured, only managed, and so it is essential that, alongside a diagnosis, healthcare professionals outline a patient’s next steps. In 2020, only 49% of respondents to a survey of RD patients reported feeling satisfied with the information provided by healthcare professionals [8]. Without accurate information from healthcare providers, individuals are left to search for information themselves, possibly leading to misinformation and worry. This is particularly difficult for young people, for whom accurate information may be less accessible.

Furthermore, while physical health is generally addressed at diagnosis, mental health is often neglected: only 23% of a 2022 survey’s respondents thought healthcare professionals considered mental and physical health equally, and almost half reported that their physician had never asked about their mental wellbeing [9]. Since studies have shown that young RD patients report a lower quality of mental health than their healthy peers, they are a particularly vulnerable group, and psychosocial support must be integrated into their treatment.

I can attest to this personally: after my first endoscopy, it was clear there was something wrong with my upper oesophagus needing further investigation. However, at this stage, achalasia had taken over my life: I struggled with motivation for everyday life, feeling disillusioned and depressed. The one thing I was looking forward to was an exchange, a term spent at a school in the USA, planned for over a year. While this delayed my next endoscopy by a month, my consultant understood that although the exchange was not the “right thing” for my condition, it was the right thing for me. Upon returning from the USA, I was ready for another gruelling period of testing and treatment, but most importantly, I had reconnected with my sense of self beyond my identity as a patient.

## Navigating uncertainty and isolation after a diagnosis

Despite my diagnosis validating months of unexplained symptoms, it was also incredibly isolating. Since I was ‘rare’, I felt that I had no one in my life who could truly relate. Without reference points of peers to compare myself to, I was afraid of my disease’s trajectory.

This isolation is particularly damaging in adolescence, a formative period in which young people are just beginning to develop their identities: it has been well documented that children with RDs report “feeling different”, regardless of whether they have a physical disability [10]. Healthcare providers can help alleviate this isolation through signposting patients to support groups: whilst I had never met another achalasia patient before Isobel, she was part of several online groups, finding comfort in their supportive communities and shared experiences. Although support groups are invaluable, offering patients emotional support and information and connecting them to others with similar experiences [11], a 2016 report of RD patients revealed that 80% of respondents were not informed of such resources by their specialist at diagnosis [12]. This is a neglected opportunity to improve young patients’ care.

## Long-term impact

Today, weeks pass without my symptoms affecting me, but I am still acutely aware of the immense impact achalasia has had on my life: although the physical symptoms were debilitating, the lasting mental toll of my condition was far more significant. Even as I write this at 19, a feat that I would not have been emotionally capable of only a few years ago, I live with a precarious uncertainty around what the future holds. However, my experience has shown me the sort of healthcare professional that, as a medical student, I hope to become. While for the foreseeable future, there will always be a knowledge gap in rare diseases, there need not be an empathy gap: by engaging with patients, professionals can shorten the diagnostic odyssey. By treating the diagnosis as a starting point, enabling access not only to physical, but also to psychosocial support, we can stop ‘rare’ meaning ‘alone’. By listening to and learning from young patients’ lived experiences, we can transform their care.

## Data Availability

Not applicable.

## Abbreviations

RD: Rare Disease
A&E: Accident and Emergency
USA: United States of America
